# Imaging-Based AI for Predicting Lymphovascular Space Invasion in Cervical Cancer: Systematic Review and Meta-Analysis

**DOI:** 10.2196/71091

**Published:** 2025-06-16

**Authors:** Lizhen She, Yunfeng Li, Hongyong Wang, Jun Zhang, Yuechen Zhao, Jie Cui, Ling Qiu

**Affiliations:** 1 Department of Radiation Oncology, The Second Hospital of Jilin University, 4026 Yatai Street, Changchun, Jilin Province, 130022, China, 86 17843128126

**Keywords:** artificial intelligence, uterine cervical neoplasms, lymphovascular space invasion, diagnostic performance, meta-analysis

## Abstract

**Background:**

The role of artificial intelligence (AI) in enhancing the accuracy of lymphovascular space invasion (LVSI) detection in cervical cancer remains debated.

**Objective:**

This meta-analysis aimed to evaluate the diagnostic accuracy of imaging-based AI for predicting LVSI in cervical cancer.

**Methods:**

We conducted a comprehensive literature search across multiple databases, including PubMed, Embase, and Web of Science, identifying studies published up to November 9, 2024. Studies were included if they evaluated the diagnostic performance of imaging-based AI models in detecting LVSI in cervical cancer. We used a bivariate random-effects model to calculate pooled sensitivity and specificity with corresponding 95% confidence intervals. Study heterogeneity was assessed using the *I*^2^ statistic.

**Results:**

Of 403 studies identiﬁed, 16 studies (2514 patients) were included. For the interval validation set, the pooled sensitivity, specificity, and area under the curve (AUC) for detecting LVSI were 0.84 (95% CI 0.79-0.87), 0.78 (95% CI 0.75-0.81), and 0.87 (95% CI 0.84-0.90). For the external validation set, the pooled sensitivity, specificity, and AUC for detecting LVSI were 0.79 (95% CI 0.70-0.86), 0.76 (95% CI 0.67-0.83), and 0.84 (95% CI 0.81-0.87). Using the likelihood ratio test for subgroup analysis, deep learning demonstrated significantly higher sensitivity compared to machine learning (*P*=.01). Moreover, AI models based on positron emission tomography/computed tomography exhibited superior sensitivity relative to those based on magnetic resonance imaging (*P*=.01).

**Conclusions:**

Imaging-based AI, particularly deep learning algorithms, demonstrates promising diagnostic performance in predicting LVSI in cervical cancer. However, the limited external validation datasets and the retrospective nature of the research may introduce potential biases. These findings underscore AI’s potential as an auxiliary diagnostic tool, necessitating further large-scale prospective validation.

## Introduction

Cervical cancer remains a predominant cause of cancer-related morbidity and mortality among women globally, with an estimated 660,000 new cases and 350,000 deaths reported in 2022 [1,2]. The prognosis of cervical cancer is determined by a variety of influencing factors, including tumor stage, histopathological type, size, lymphovascular space invasion (LVSI), and the presence of distant metastasis [3-5]. Among these, LVSI is a critical prognostic factor, as its presence is associated with an increased risk of lymph node metastasis, disease recurrence, and poorer overall survival [6]. It refers to the infiltration of cancer cells into the lymphatic system or blood vessels [6]. Researchers have demonstrated that LVSI is linked to worse survival outcomes. According to Marchiolé et al [7], the incidence of LVSI was 2-fold higher in patients with cervical cancer recurrence than in those without recurrence. Pol et al [8] demonstrated that patients without LVSI exhibited significantly superior outcomes in both disease-free survival and overall survival.

The presence of LVSI significantly impacts treatment decisions in cervical cancer [9]. Patients with LVSI-positive status often necessitate multimodal therapy, including radical surgery with lymphadenectomy combined with chemotherapy or radiotherapy, with the sequence determined by tumor stage and risk factors [10]. Current diagnostic methods for cervical cancer include imaging techniques such as computed tomography (CT), positron emission tomography/computed tomography (PET/CT), and magnetic resonance imaging (MRI), which are used to evaluate LVSI. However, these imaging modalities have limitations in accurately detecting LVSI. Based on findings from Holtz and Dunton [11] and Zhu et al [12], CT demonstrated inferior soft tissue visualization and diagnostic performance in assessing cervical cancer tumor invasion compared to MRI. Specifically, PET/CT exhibits inherent constraints in soft tissue resolution, limiting its diagnostic performance for detecting minute neoplastic lesions [13,14]. According to Park et al [15], among stage IB1 cervical cancer patients with no visible tumors on MRI, 4.7% still exhibited LVSI, underscoring the limitations of MRI in detecting microscopic features. Similarly, Woo et al [16] reported that MRI’s diagnostic rate for postconization residual tumors was 32.7%, significantly lower than the actual occurrence rate of 54.5%, highlighting its restricted sensitivity in detecting residual disease. These limitations demonstrate the need for advanced diagnostic tools, such as artificial intelligence (AI), which can enhance diagnostic accuracy by detecting subtle imaging patterns and providing quantitative, reproducible analyses that surpass human interpretation.

Consequently, there is growing interest in applying image-based AI to improve the accuracy of LVSI detection. AI-based diagnostic tools have demonstrated variable performance in predicting LVSI. Some studies show high performance with area under the curve (AUC) values of 0.94 and 0.923, respectively [17,18]. In contrast, other studies, such as Li et al [19] and Wang et al [20], observed considerably lower performance, with AUC values of 0.72 and 0.73, respectively. These discrepancies can be attributed to factors such as data quality, sample size, and model architecture. Low-quality datasets, such as retrospective studies or single-center studies, may introduce selection bias and limit the generalizability of models, thereby affecting the reliability of radiomics approaches in clinical practice [21]. Additionally, smaller or less diverse sample sizes also restrict model generalizability [22]. These discrepancies highlight the need for further research to assess the reliability and external validation of AI models in clinical practice.

Given the limitations of conventional imaging modalities and the inconsistent performance of AI tools, our systematic review and meta-analysis aim to comprehensively evaluate the diagnostic accuracy of imaging-based AI in predicting LVSI in cervical cancer, addressing critical gaps in current literature.

## Methods

This meta-analysis was conducted in strict accordance with the Preferred Reporting Items for Systematic Reviews and Meta-Analyses of Diagnostic Test Accuracy (PRISMA-DTA) guidelines [23].

### Search Strategy

A systematic literature search was conducted in PubMed, Embase, and Web of Science without date restrictions to identify relevant studies published up to November 9, 2024. The search strategy included 4 groups of terms: those related to AI (eg, “artificial intelligence,” “machine learning,” “deep learning”), cervical cancer (eg, “uterine cervical neoplasms,” “cervix,” “cervical”), lymph-vascular space invasion (eg, “LVSI,” “lymphatic vessel invasion,” “lymphatic permeation”) and imaging modalities (eg, “MRI,” “magnetic resonance imaging,” “PET/CT,” “positron emission tomography-computed tomography”). Both keywords and Medical Subject Headings terms were used, and complete strategy could be seen in Multimedia Appendix 1. We manually reviewed the reference lists of selected studies for additional articles and repeated the search on December 11, 2024, to ensure the inclusion of recent publications.

### Inclusion and Exclusion Criteria

The selection of studies adhered to the PICOS (Population, Intervention, Comparison, Outcome, Study Design) framework to ensure rigorous assessment and relevance.

- Population (P): Studies involved adult patients diagnosed with cervical cancer who underwent LVSI assessment.
- Intervention (I): Imaging-based AI models, including CT, MRI, ultrasound, and PET/CT, were used for LVSI evaluation.
- Comparison (C): No diagnostic tool was used as a comparator.
- Outcome (O): Studies providing quantitative diagnostic performance metrics, including sensitivity, specificity, and AUC, with extractable true positive (TP), true negative (TN), false positive (FP), and false negative (FN) data.
- Study design (S): Only studies published in English and focused on pretreatment prediction models were included.

Studies were excluded if they met any of the following conditions: (1) irrelevant titles or abstracts; (2) focused solely on lymph node metastasis rather than LVSI; (3) inappropriate article types, including reviews, conference abstracts, case reports, or meta-analyses; case reports were excluded due to their limited sample size, typically involving only a single patient, which makes them unsuitable for statistical analysis or model evaluation; (4) studies with incomplete or uninterpretable data, where TP, FP, FN, and TN values for internal and external validation sets could not be extracted; and (5) animal or in vitro studies. The screening process was conducted in duplicate by 2 independent reviewers (LS and YL), who first evaluated titles and abstracts for relevance. Full-text articles were then assessed against the inclusion and exclusion criteria. Duplicate articles were identified and removed using EndNote’s (Clarivate) duplicate detection tool, followed by manual verification. Discrepancies between reviewers during the screening process were resolved through discussion, and if consensus could not be reached, a third reviewer (HW) was consulted to make the final decision.

### Quality Assessment

To comprehensively assess the quality of included studies, we adapted a recognized tool, the revised Quality Assessment of Diagnostic Accuracy Studies-2 (QUADAS-2) [24], by replacing certain criteria with more relevant ones from the Prediction Model Risk of Bias Assessment Tool (PROBAST) [25]. The rationale for this adaptation lies in the complementary strengths of the 2 tools. QUADAS-2 is widely recognized for evaluating the risk of bias in diagnostic accuracy studies, but it does not fully address methodological considerations specific to prediction models, such as variable selection, outcome definitions, or overfitting. PROBAST, on the other hand, is specifically designed to assess risk of bias in prediction model studies. By combining elements from both tools, we tailored the modified QUADAS-2 tool to account for the unique characteristics of studies exploring image-based AI models for LVSI detection.

Our modified QUADAS-2 tool evaluates 4 domains: participants, index test (AI algorithm), reference standard, and analysis. In addition to assessing the risk of bias across each domain, we also evaluated applicability concerns in the first 3 domains. Two independent reviewers applied the modified QUADAS-2 tool to assess the risk of bias in each study, resolving any disagreements through discussion.

### Data Extraction

Two independent reviewers assessed study eligibility and performed data extraction, resolving any discrepancies through consensus, with a third reviewer serving as an adjudicator if needed. Data extraction encompassed the primary author’s name, publication year, study design, and country of origin. Additionally, key elements such as imaging modality, reference standard, and total and LVSI+ counts of patients, lesions, or images in training, internal validation, and external validation sets were recorded. Further details included age, AI method, AI model algorithms, and performance outcomes, specifically the TP, FP, TN, and FN counts for both internal and external validation sets. Internal validation refers to the evaluation of model performance using a subset of data from the same source as the training data. External validation, in contrast, involves using an independent dataset that originates from a different source than the training set.

### Outcome Measures

The principal outcome measures used in this analysis were sensitivity, specificity, and AUC for both internal and external validation sets. Sensitivity was defined as the proportion of TP scans among the total number of positive cases (TP+FN) for patients, images, or lesions. Specificity was defined as the proportion of true negative (TN) scans among the total number of negative cases (TN+FP). The AUC, representing the area under the receiver operating characteristic curve, served as a summary measure of the model’s diagnostic ability. We extracted AI performance data from internal validation sets and external validation sets. For studies reporting multiple machine learning or deep learning algorithms, only the best diagnostic performance model or algorithm (with the highest AUC value) was included as a representative of the study in the meta-analysis.

### Statistical Analysis

A bivariate random-effects analytical approach was used to generate pooled estimations of sensitivity and specificity outcomes for AI-based assessments of LVSI in both internal and external validation sets, accompanied by 95% CIs. We used a summary receiver operating characteristic model to generate the summary receiver operating characteristic curve and calculate the AUC. Additionally, Fagan plots were created to provide a visual representation of the clinical utility of the models.

To assess heterogeneity among studies, we calculated the *I*² statistic, interpreting values of 25%, 50%, and 75% as indicating low, moderate, and high heterogeneity, respectively. For internal validation sets, we performed subgroup analyses by the number of patients (>150 vs ≤150), region (single vs multiple centers), AI method (deep learning vs machine learning), AI algorithm (logistic regression vs support vector machine), and imaging modality (MRI vs PET/CT). Publication bias was evaluated using Deeks’ funnel plot. All statistical analyses were conducted in Stata 15.1, with significance set at *P*<.05. Risk of bias for study quality was assessed using RevMan 5.4 (The Cochrane Collaboration) for comprehensive risk of bias assessment.

## Results

### Study Selection

**Figure 1.**
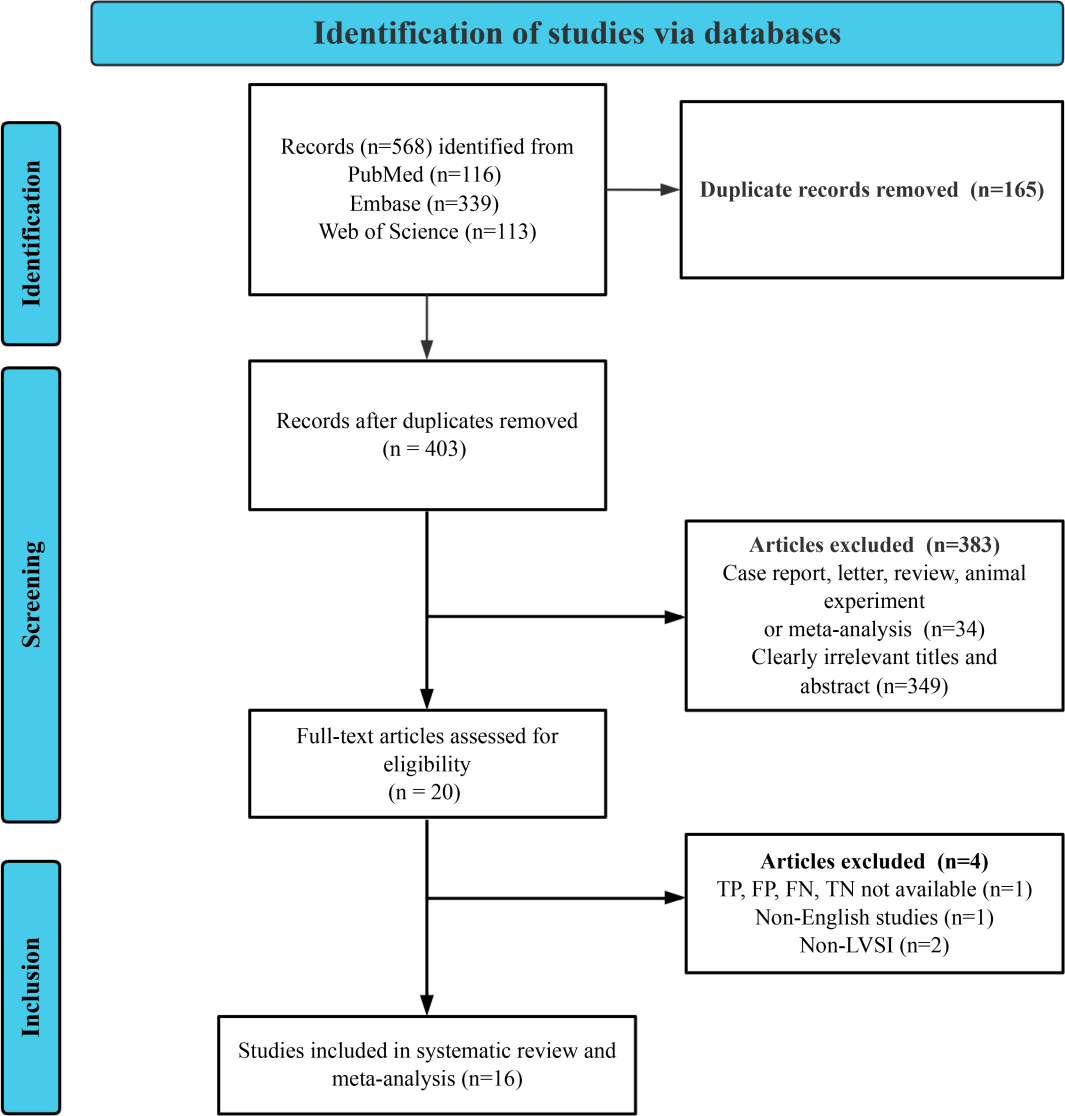
Flow chart of the screening process for included studies. TP: true positive; FP: false positive; FN: false negative; TN: true negative; LVSI: lymphovascular space invasion.

A systematic literature retrieval and analysis was methodically executed across 3 authoritative databases: PubMed, Embase, and Web of Science, yielding 568 studies. After removing 165 duplicates, 383 studies were excluded based on initial screening criteria. Subsequently, 20 full-text articles were further assessed. In total, 4 studies were excluded due to unavailable data (TP, TN, FP, and FN, n=1), non-English language (n=1), or not focusing on LVSI (n=2). Ultimately, 16 studies met all inclusion criteria and were included in the final analysis [17-20,26-37]. The PRISMA (Preferred Reporting Items for Systematic Reviews and Meta-Analyses) flowchart detailing this selection process is shown in Figure 1.

### Study Description and Quality Assessment

This meta-analysis included 16 studies, all of which included an internal validation set with a total of 1092 participants (ranging from 25 to 198 participants per study). In total, 4 studies included an external validation set [29,30,32,37], involving 203 participants (ranging from 26 to 102 participants). In total, 15 studies used MRI as the imaging modality, while 1 study used PET/CT [37]. Machine learning techniques were applied in 15 studies, with 1 study using deep learning [27]. Among the AI model algorithms, LR was the most common AI model algorithm, used in 9 studies [17,19,28,29,32-35,37], followed by support vector machine (SVM) in 5 studies [18,20,30,31,36], and decision tree and convolutional neural network algorithms, each used in 1 study [26,27]. The gold standard for diagnosis was all pathological examination. All studies were retrospective and conducted in China. Study and patient characteristics, as well as technical features, are summarized in Tables 1-3.

**Figure 2.**
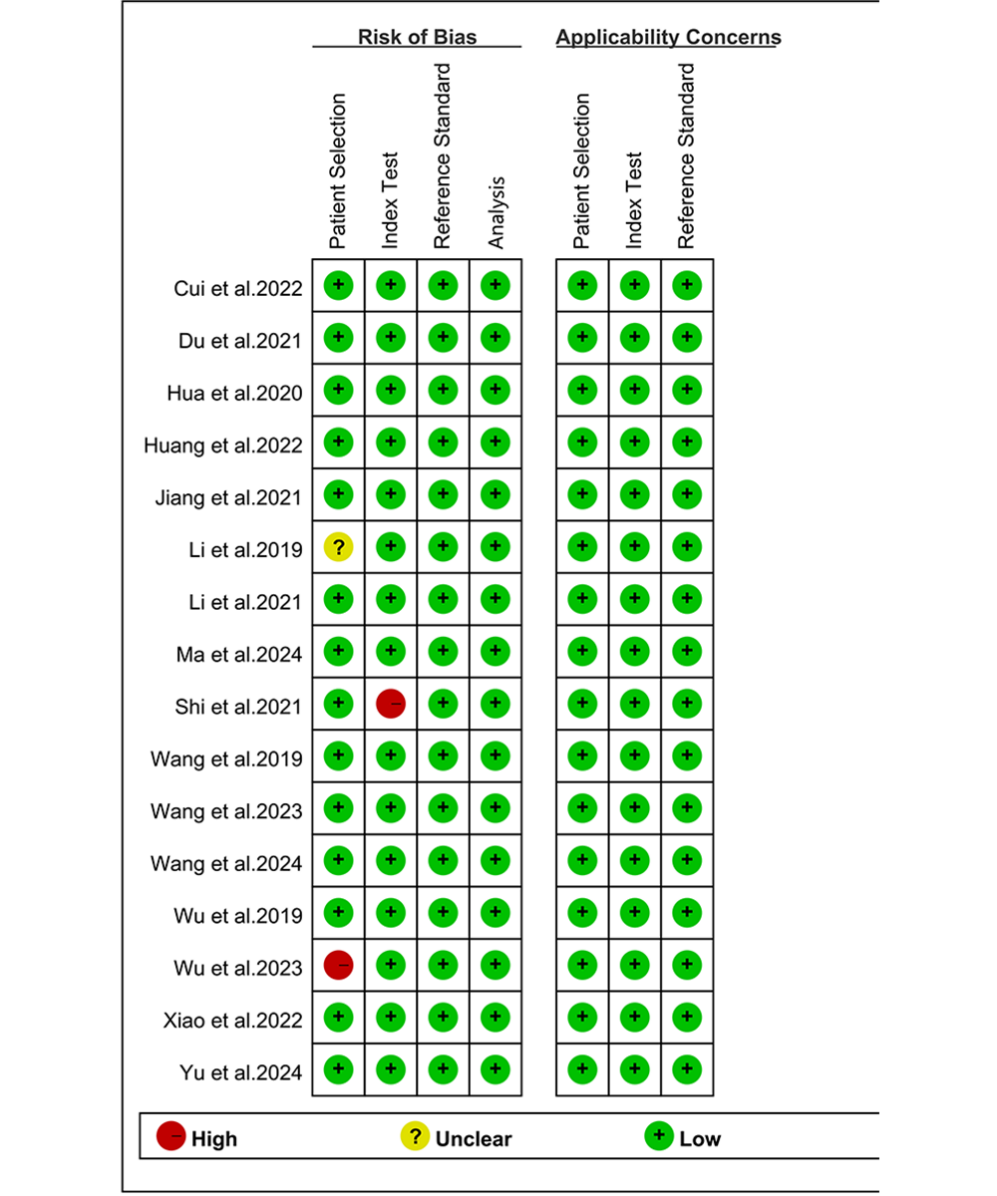
Risk of bias and applicability assessment of included studies using the revised Quality Assessment of Diagnostic Accuracy Studies-2 (QUADAS-2) tool [9,17,18,20,26-37].

Quality assessment was performed using the QUADAS-2 revised tool, as shown in Figure 2 [9,17,18,20,26-37]. For patient selection, one study was classified as “high risk” due to inappropriate exclusion criteria that may omit specific populations, such as those with a history of radiotherapy or chemotherapy [29]. One study [19] was rated as “unclear” because it was uncertain whether the patients were included consecutively. Regarding the index test in risk of bias, one study was rated as “high risk” for inadequate reporting of model training and evaluation processes [32]. Overall, the quality of the included studies was deemed acceptable.

**Table 1.** Study and patient characteristics of the included studies.

Author	Year	Country	Study design	Imaging modality	Reference standard
Yu et al [26]	2024	China	Retro^a^	MRI^b^	Pathology
Li et al [9]	2019	China	Retro	MRI	Pathology
Jiang et al [27]	2021	China	Retro	MRI	Pathology
Wu et al [28]	2019	China	Retro	MRI	Pathology
Wu et al [29]	2023	China	Retro	MRI	Pathology
Wang et al [30]	2023	China	Retro	MRI	Pathology
Hua et al [31]	2020	China	Retro	MRI	Pathology
Shi et al [32]	2021	China	Retro	MRI	Pathology
Huang et al [17]	2022	China	Retro	MRI	Pathology
Xiao et al [33]	2022	China	Retro	MRI	Pathology
Cui et al [34]	2022	China	Retro	MRI	Pathology
Ma et al [35]	2024	China	Retro	MRI	Pathology
Wang et al [36]	2024	China	Retro	MRI	Pathology
Li et al [37]	2021	China	Retro	PET/CT^c^	Pathology
Du et al [18]	2021	China	Retro	MRI	Pathology
Wang et al [20]	2019	China	Retro	MRI	Pathology

a Retro: retrospective.

b MRI: magnetic resonance imaging.

c PET/CT: positron emission tomography-computed tomography.

**Table 2.** Patient characteristics of included studies.

Author	Year	Patients, lesions, or images per set			Age	Number of LVSI^a^+ patients, lesions, or images
		Training	Internal validation	External validation		
Yu et al [26]	2024	120	60	NR^b^	Training: median 53 (range 47-57) Internal validation: median 50 (range 46-54)	Training: 46 Internal validation: 27
Li et al [19]	2019	70	35	NR	Training: LVSI+: mean 49.34 (SD 8.55); non-LVSI: mean 46.54 (SD 9.66) Internal validation: LVSI+: mean 54.54 (SD 10.42); non-LVSI: mean 48.59 (SD 10.69)	Training: 29 Internal validation: 13
Jiang et al [27]	2021	2056	2056	NR	Median 50.42 (range 27-70)	Training: 862 Internal validation: 862
Wu et al [28]	2019	56	56	NR	Median 50 (range 29‐67)	Training: 31 Internal validation: 31
Wu et al [29]	2023	129	129	39	Training: LVSI+: mean 48.07 (SD 10.16); non-LVSI: mean 53.18 (SD 9.47) Internal validation: LVSI+: mean 48.07 (SD 10.16); non-LVSI: mean 53.18 (SD 9.47) External validation: LVSI+: mean 52.08 (SD 12.24); non-LVSI: mean 52.81 (SD 9.46)	Training: 46 Internal validation: 46 External validation: 12
Wang et al [30]	2023	198	198	102	Training: LVSI+: mean 51.35 (SD 10.49); non-LVSI: mean 51.63 (SD 10.84) Internal validation: LVSI+: mean 51.35 (SD 10.49); non-LVSI: mean 51.63 (SD 10.84) External validation: LVSI+: mean 49.06 (SD 10.52); non-LVSI: mean 51.81 (SD 8.73)	Training: 104 Internal validation: 104 External validation: 54
Hua et al [31]	2020	111	56	NR	Training: LVSI+: mean 49.90 (SD 10.06); non-LVSI: mean 51.34 (SD 8.73) Internal validation: LVSI+: mean 50.57 (SD 7.57); non-LVSI: mean 52.67 (SD 10.04)	Internal validation: 23 Training: 44
Shi et al [32]	2021	160	44	36	Training: LVSI+: mean 49.97 (SD 9.78); non-LVSI: mean 52.68 (SD 8.72) Internal validation: LVSI+: mean 54.62 (SD 9.07); non-LVSI: mean 49.53 (SD 9.85) External validation: LVSI+: mean 50.21 (SD 12.67); non-LVSI: mean 55.36 (SD 10.36)	Training: 65 Internal validation: 16 External validation: 14
Huang et al [17]	2022	100	25	NR	Total: mean 47.94 (SD 9.01); LVSI+: mean 47.23 (SD 8.23); non-LVSI: mean 48.20 (SD 9.31)	Training: 29 Internal validation: 5
Xiao et al [33]	2022	154	79	NR	Training: mean 50.0 (SD 9.3) Internal validation: mean 49.9 (SD 10.8); LVSI+: mean 49.5 (SD 10.1); non-LVSI: mean 50.9 (SD 9.2)	Training: 106 Internal validation: 45
Cui et al [34]	2022	108	55	NR	Training: LVSI+: mean 49.42 (SD 9.16); non-LVSI: mean 51.11 (SD 9.43) Internal validation: LVSI+: mean 52.23 (SD 9.75); non-LVSI: mean 52.94 (SD 9.03)	Training: 43 Internal validation: 22
Ma et al [35]	2024	86	38	NR	Training: LVSI+: median 52 (range 47.2‐57.5); Non-LVSI: median 57.5 (range 51.2‐64.8) Internal validation: LVSI+: mean 53.2 (SD 6.8); non-LVSI: mean 53.4 (SD 12.7)	Training: 24 Internal validation: 8
Wang et al [36]	2024	61	40	NR	LVSI+: mean 53.25 (SD 9.72); Non-LVSI: mean 53.57 (SD 10.36)	Training: 34 Internal validation: 14
Li et al [37]	2021	61	25	26	Training: median 50 (range 33-74) Internal validation: median 51 (range 40-58) External validation: median 52 (range 40-74)	Training: 30 Internal validation: 12 External validation: 15
Du et al [18]	2021	104	45	NR	Training: LVSI+: median 45 (range 37-53); non-LVSI: median 48 (range 40-56) Internal validation: LVSI+: median 44 (range 35-53); non-LVSI: median 46 (range 42-50)	Training: 45 Internal validation: 22
Wang et al [20]	2019	80	40	NR	Training: median 49.20 (range 29-67) Internal validation: mean 50.45 (SD 32-75)	Internal validation: 7 Training: 25

a LVSI: lymphovascular space invasion.

b NR not reported.

**Table 3.** Technical aspects of included studies.

Author	Year	AI^a^ method	AI model algorithms	Interval validation sets				External validation sets			
				TP^b^	FP^c^	FN^d^	TN^e^	TP	FP	FN	TN
Yu et al [26]	2024	Machine learning	DT^f^	22	9	5	24	NR^g^	NR	NR	NR
Li et al [9]	2019	Machine learning	LR^h^	9	5	4	17	NR	NR	NR	NR
Jiang et al [27]	2021	Deep learning	CNN^i^	759	296	103	898	NR	NR	NR	NR
Wu et al [28]	2019	Machine learning	LR	27	7	4	18	NR	NR	NR	NR
Wu et al [29]	2023	Machine learning	LR	34	7	12	76	10	5	2	22
Wang et al [30]	2023	Machine learning	SVM^j^	81	16	23	78	40	12	14	36
Hua et al [31]	2020	Machine learning	SVM	17	11	6	22	NR	NR	NR	NR
Shi et al [32]	2022	Machine learning	LR	14	8	2	20	13	7	1	15
Huang et al [17]	2022	Machine learning	LR	5	6	0	14	NR	NR	NR	NR
Xiao et al [33]	2022	Machine learning	LR	36	8	9	26	NR	NR	NR	NR
Cui et al [34]	2022	Machine learning	LR	17	9	5	24	NR	NR	NR	NR
Ma et al [35]	2024	Machine learning	LR	6	7	2	23	NR	NR	NR	NR
Wang et al [36]	2024	Machine learning	SVM	13	9	1	17	NR	NR	NR	NR
Li et al [37]	2021	Machine learning	LR	8	0	4	13	12	2	3	9
Du et al [18]	2021	Machine learning	SVM	18	3	4	20	NR	NR	NR	NR
Wang et al [20]	2019	Machine learning	SVM	6	12	1	21	NR	NR	NR	NR

a AI: artificial intelligence.

b TP: true positive.

c FP: false positive.

d FN: false negative.

e TN: true negative.

f DT: decision tree.

g NR: not reported.

h LR: logistic regression.

i CNN: convolutional neural network.

j SVM: support vector machine.

### Diagnostic Performance of Internal Validation Sets and External Validation Sets for Artificial Intelligence in Predicting Lymph Vascular Space Invasion of Cervical Cancer

**Figure 3.**
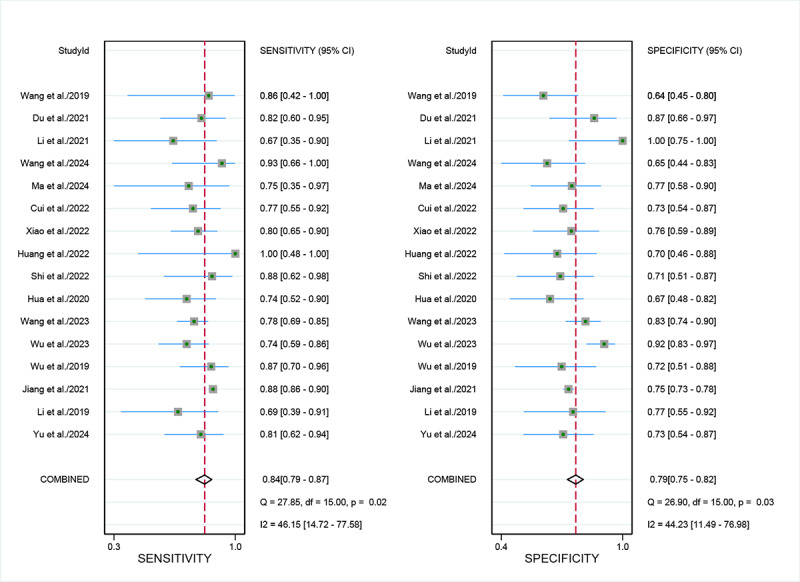
Forest plot of sensitivity and specificity for artificial intelligence–based lymphovascular space invasion diagnosis in cervical cancer: internal validation set [9,17,18,20,26-37].

**Figure 4.**
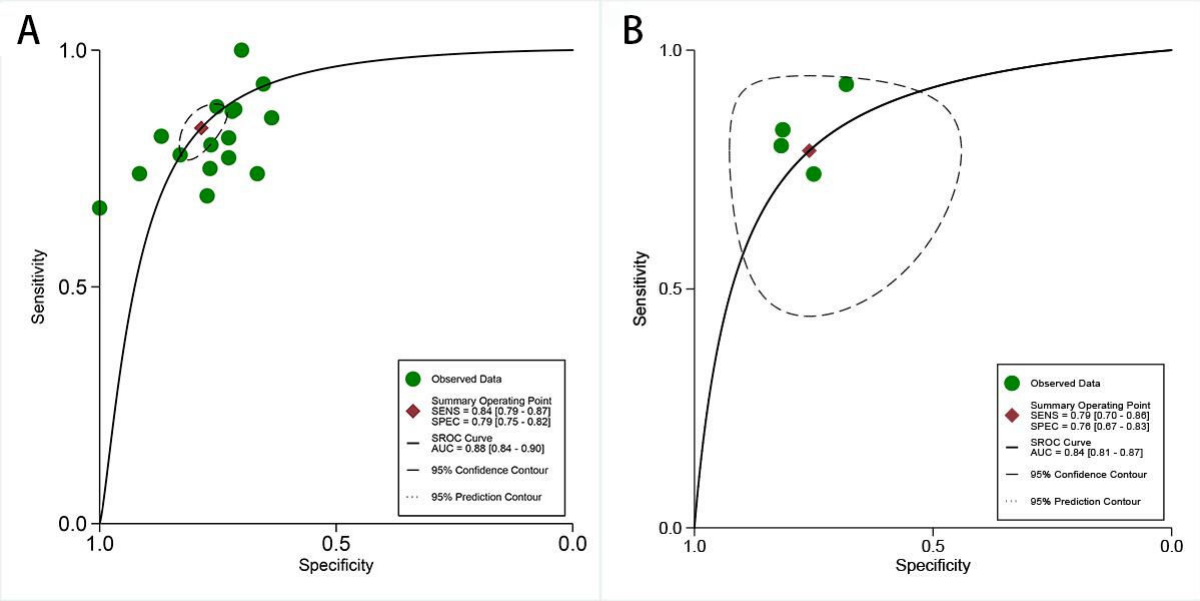
Receiver operating characteristic (ROC) curves for artificial intelligence–based lymphovascular space invasion prediction in cervical cancer: (**A**) internal validation set and (**B**) external validation set. AUC: area under the curve; SENS: sensitivity; SPEC: specificity; SROC: summary receiver operating characteristic.

**Figure 5.**
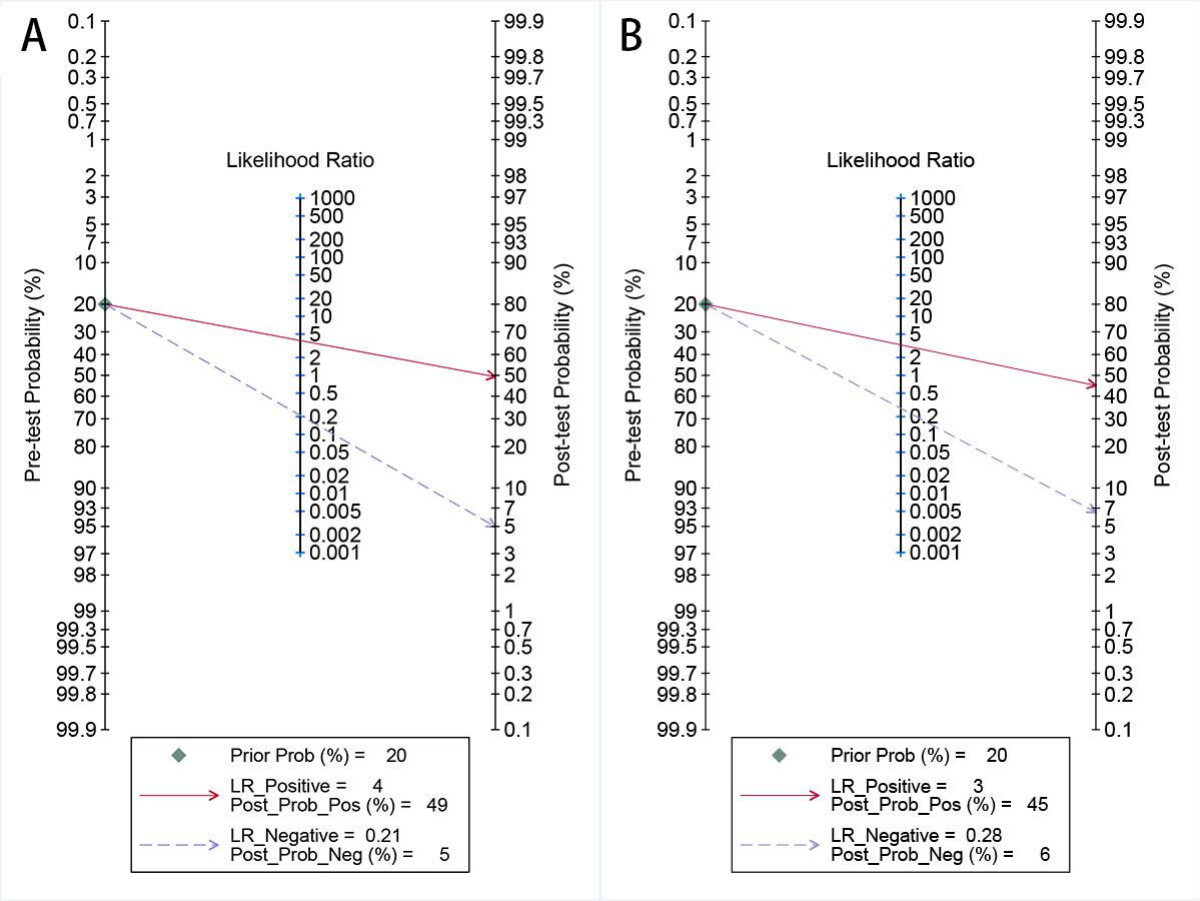
Fagan’s nomograms for artificial intelligence–based lymphovascular space invasion diagnostic performance in cervical cancer: (**A**) internal validation set and (**B**) external validation set. LR: logistic regression.

For internal validation sets, the sensitivity in detecting LVSI of cervical cancer was 0.84 (95% CI 0.79‐0.87), and the specificity was 0.79 (95% CI 0.75‐0.82; Figure 3 [9,17,18,20,26-37]), with an AUC of 0.88 (95% CI 0.84‐0.90; Figure 4A). Using a pretest probability of 20%, the Fagan nomogram demonstrates a positive likelihood ratio of 49% and a negative likelihood ratio of 5% (Figure 5A).

**Figure 6.**
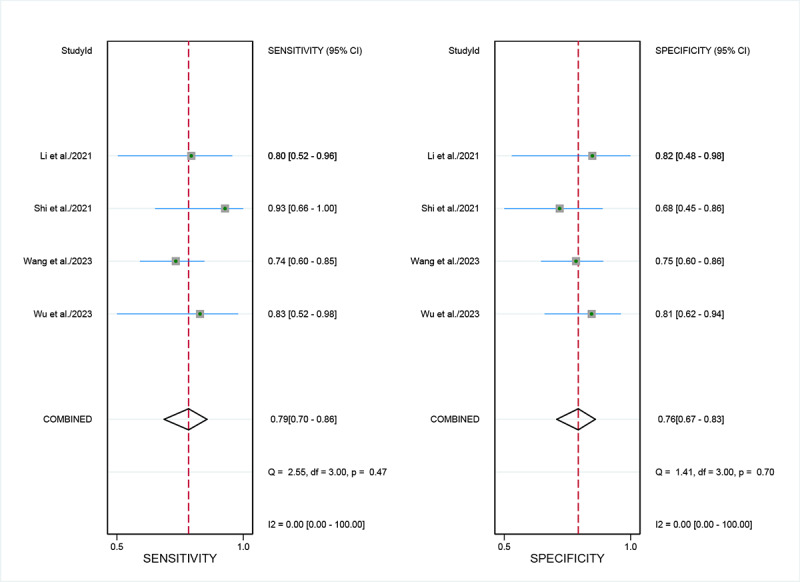
Forest plot of external validation set for sensitivity and specificity of artificial intelligence in diagnosing lymphovascular space invasion in cervical cancer [29,30,32,37].

For external validation sets, the sensitivity in detecting LVSI was 0.79 (95% CI 0.70‐0.86), and the specificity was 0.76 (95% CI 0.67‐0.83; Figure 6 [29,30,32,37]), with an AUC of 0.84 (95% CI 0.81‐0.87; Figure 4B). Using a pretest probability of 20%, the Fagan nomogram demonstrates a positive likelihood ratio of 45% and a negative likelihood ratio of 6% (Figure 5B).

### Subgroup Analysis of Internal Validation Sets of Image-Based AI for Lymphovascular Space Invasion in Cervical Cancer

In the subgroup analysis, we evaluated diagnostic performance across the number of internal validation patients, region, AI methods, AI algorithms, imaging modalities, and data source (Table 4). Number of internal validation patients (>150 vs ≤150) showed no statistically significant differences in sensitivity (*P*=.35) and specificity (*P*=.08). Single-center studies demonstrated significantly different specificities compared to multicenter studies (*P*<.001). Deep learning (1 study) exhibited higher sensitivity compared to machine learning (15 studies, *P*=.01), with no significant differences in specificity (*P*=.29). Among AI algorithms, LR (9 studies) and SVM (5 studies) showed comparable diagnostic performance, with no significant differences in sensitivity (*P*=.77) or specificity (*P*=.36). Imaging modality analysis revealed a significant difference in sensitivity between MRI (15 studies) and PET/CT (1 study, *P*=.01), while specificity remained consistent (*P*=.29). The radiomic model showed a higher sensitivity, and the radiomic and clinical model showed higher specificity; the differences were statistically significant (*P*=.05 for both sensitivity and specificity comparisons).

**Table 4.** Subgroup analysis of imaging-based artificial intelligence performance in internal validation cohorts for lymphovascular space invasion detection in cervical cancer.

Subgroup	Studies, n	Sensitivity (95% CI)	Subgroup difference *P* value	Specificity (95% CI)	Subgroup difference *P* value
Number of internal validation patients			.35		.08
>150	8	0.81 (0.75‐0.86)		0.80 (0.75‐0.84)	
≤150	8	0.84 (0.78‐0.88)		0.73 (0.65‐0.79)	
Region			.09		<.001
Single center	12	0.84 (0.79‐0.89)		0.74 (0.70‐0.78)	
Multiple centers	4	0.77 (0.69‐0.84)		0.85 (0.80‐0.89)	
AI^a^ method			.01		.29
Deep learning	1	0.88 (0.86‐0.90)		0.75 (0.73‐0.78)	
Machine learning	15	0.79 (0.75‐0.83)		0.78 (0.74‐0.81)	
AI algorithms			.77		.36
LR^b^	9	0.79 (0.72‐0.84)		0.79 (0.73‐0.84)	
SVM^c^	5	0.80 (0.73‐0.86)		0.75 (0.66‐0.81)	
Imaging-based			.01		.29
PET/CT^d^	1	0.88 (0.86‐0.90)		0.75 (0.73‐0.78)	
MRI^e^	15	0.79 (0.75‐0.83)		0.78 (0.74‐0.81)	
Data source			.05		.05
Radiomic	7	0.86 (0.81‐0.90）		0.75 (0.72‐0.78）	
Radiomic and clinical	9	0.78 (0.71‐0.83）		0.81 (0.76‐0.85）	

a AI: artificial intelligence.

b LR: logistic regression.

c SVM: support vector machine.

d PET/CT: positron emission tomography-computed tomography.

e MRI: magnetic resonance imaging.

### Publication Bias

**Figure 7.**
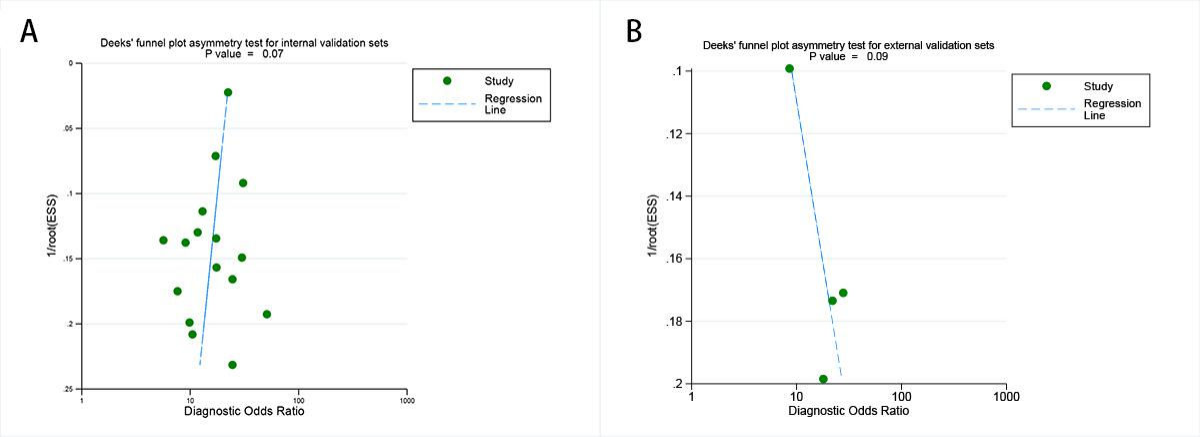
Deeks’ funnel plots for publication bias assessment in artificial intelligence–based lymphovascular space invasion prediction: (**A**) internal validation set and (**B**) external validation set.

Deeks’ funnel plot asymmetry test showed that there is no significant publication bias both for the internal validation sets (*P*=.07; Figure 7A) and external validation sets (*P*=.09; Figure 7B) for AI.

## Discussion

### Principal Findings

Based on our comprehensive literature review, this is the first meta-analysis comprehensively evaluating imaging-based AI for detecting LVSI in cervical cancer. The pooled sensitivity, specificity, and AUC in our analysis for interval validation were 0.84, 0.79, and 0.88, respectively, while for external validation, these metrics were slightly lower at 0.79, 0.76, and 0.84. The slightly lower performance in external validation compared to internal validation is expected when applying AI models to independent datasets. This decline may be due to differences in patient populations, imaging protocols, data quality, and clinical practices across institutions. Despite this, the proposed AI models still show reasonable generalizability. They demonstrate potential for robust performance in diverse datasets and clinical settings.

Deep learning showed superior sensitivity (0.88) compared to machine learning (0.79), likely due to its advanced capabilities in processing complex imaging data. Deep learning models, particularly convolutional neural networks, excel at autonomously extracting hierarchical features from imaging datasets, revealing subtle patterns that manual feature extraction often misses [27]. And by integrating diverse imaging variables like tissue texture, morphological characteristics, and intensity variations, deep learning enables more precise LVSI-positive case differentiation [27,38,39]. However, given the limited number of deep learning studies, the observed higher sensitivity may be insufficiently generalizable. Larger-scale, multicenter studies are essential to validate the potential superiority of deep learning approaches in LVSI detection. Additionally, deep learning is challenged by high overfitting risk and significant computational demands, which can hinder its practical application in clinical settings [27,40].

### Comparison to Prior Work

Comparative imaging analysis of LVSI detection revealed differential sensitivity across modalities. MRI-based AI demonstrated a pooled sensitivity of 0.79, whereas PET/CT-based AI reported a marginally superior sensitivity of 0.88. The potential enhanced diagnostic performance of PET/CT can be attributed to its sophisticated capacity for integrating functional and anatomical data, thereby facilitating more nuanced metabolic and structural characterization [41]. However, it is important to note that only 1 study used PET/CT in this analysis, and the limited sample size may have influenced the reported sensitivity. This warrants cautious interpretation of the results and highlights the need for further studies with larger PET/CT-based datasets to confirm these findings.

In 2024, Zhang et al [22] conducted a meta-analysis specifically examining MRI-based radiomics models for predicting LVSI in cervical cancer. Their findings reported a sensitivity of 0.79, specificity of 0.73, and an AUC of 0.83. In contrast, our study achieved superior results in the internal validation set, with a sensitivity of 0.84, specificity of 0.79, and an AUC of 0.88. The improved performance in our research could be attributed to the incorporation of more recent studies and a larger sample size, which likely enhanced the accuracy and robustness of the model. Similarly, a recent meta-analysis by Zhao et al [21] reported comparable diagnostic performance metrics, with a sensitivity of 0.83, specificity of 0.74, and an AUC of 0.86. Unlike their studies focusing only on MRI, our meta-analysis integrated multiple imaging modalities, including MRI and PET/CT, thus offering a more comprehensive evaluation of AI-based diagnostic approaches. Moreover, we pioneered the approach of stratifying the analysis into internal and external validation cohorts, enabling a more rigorous evaluation of the models’ diagnostic performance and generalizability. The slight decline in external validation cohorts indicates acceptable generalizability and provides clinicians with realistic expectations regarding model performance in real-world clinical scenarios.

### Heterogeneity

Our meta-analysis demonstrated no significant heterogeneity in both internal and external validation datasets. However, the Deeks’ funnel plot revealed a borderline *P* value of approximately 0.07, suggesting the potential presence of publication bias. Therefore, we used a bivariate random-effects model for sensitivity and specificity pooling, acknowledging inherent clinical heterogeneity and potential bias. Subgroup analyses were strategically conducted based on the number of patients, region, AI method, AI algorithms, imaging modalities, and data source. The results indicated that both the region and the AI methods significantly influenced the pooled outcomes. However, several additional factors may also contribute to clinical heterogeneity, including variations in AI algorithm architectures, hyperparameter optimization strategies, image acquisition protocols, preprocessing techniques, feature selection methods, and discrepancies in LVSI assessment criteria among pathologists. As reported in Huang et al [17], variations in image acquisition protocols, such as differences in imaging resolution or contrast enhancement methods, may impact the diagnostic performance. Similarly, heterogeneity arising from AI algorithm design, such as the use of different convolutional neural network algorithms, has been shown to influence overall results [27].

### Future Directions

Despite these challenges, our findings revealed promising diagnostic performance of AI-based approaches for LVSI detection in cervical cancer, suggesting their potential to streamline clinical workflows and enhance diagnostic accuracy. The implementation of AI-driven approaches in cervical cancer surveillance has increased over the years, yielding encouraging therapeutic prospects [42]. The implementation of imaging-based AI tools could particularly benefit primary health care systems, especially in resource-limited settings where specialist expertise may be scarce. However, challenges include limited computing infrastructure, data storage constraints, and the need for tailored training programs [43]. Additionally, AI systems could optimize screening efficiency and support treatment planning decisions [44]. There is the lack of direct comparisons between the performance of AI models and that of radiologists. Such comparisons are critical to understanding the relative strengths and weaknesses of AI in clinical practice. Future studies should aim to investigate head-to-head comparisons between AI algorithms and radiologists.

### Limitations

There are several limitations in our meta-analysis. First, external validation was performed in only 4 of 16 studies, raising concerns about model generalizability. The limited external validation increases the risk of overfitting, where models may perform exceptionally well on training datasets but demonstrate reduced performance on independent datasets. Second, the inherently retrospective design of the included studies may introduce selection biases and limitations in data collection. Second, all studies were conducted in China, which raises concerns about the generalizability of our findings to other populations and health care settings. These factors underscore the critical need for future prospective investigations that not only address the biases associated with retrospective designs but also validate and strengthen the robustness of our findings across diverse demographics and clinical environments. Third, the absence of direct comparative analyses between AI and radiologist interpretations represents a significant research gap; further comparisons are needed.

In conclusion, imaging-based AI, particularly deep learning algorithms, demonstrates promising diagnostic performance in predicting LVSI in cervical cancer. However, the limited external validation datasets and the retrospective nature of the research may introduce potential biases. These findings underscore AI’s potential as an auxiliary diagnostic tool, necessitating further large-scale prospective validation.

## Supplementary material

10.2196/71091 Multimedia Appendix 1 Additional materials.

10.2196/71091 Checklist 1 PRISMA (2020) checklist.

## Abbreviations

AI: artificial intelligence
AUC: area under the curve
CT: computed tomography
FN: false negative
FP: false positive
LVSI: lymphovascular space invasion
MRI: magnetic resonance imaging
PET/CT: positron emission tomography/computed tomography
PICOS: Population, Intervention, Comparison, Outcome, Study Design
PRISMA: Preferred Reporting Items for Systematic Reviews and Meta-Analyses
PRISMA-DTA: Preferred Reporting Items for Systematic Reviews and Meta-Analyses of Diagnostic Test Accuracy
PROBAST: Prediction Model Risk of Bias Assessment Tool
QUADAS-2: Quality Assessment of Diagnostic Accuracy Studies-2
SVM: support vector machine
TN: true negative
TP: true positive
