# Supplementary material for: Imaging-Based AI for Predicting Lymphovascular Space Invasion in Cervical Cancer: Systematic Review and Meta-Analysis
Source: J Med Internet Res. 2025 Jun 16;27:e71091. doi: 10.2196/71091 (PMC12185032; doi:10.2196/71091)
Supplement: Multimedia Appendix 1 [file jmir-v27-e71091-s001.docx]

**Supplementary Table 1: Detailed search strategy and keywords used for database retrieval.**

| Database | Search strategy |
| --- | --- |
| PubMed | ("Artificial Intelligence"[Mesh] OR "Machine Learning"[Mesh] OR "Deep Learning"[Mesh] OR "Artificial Intelligence"[Title/Abstract] OR "AI"[Title/Abstract] OR "Machine Learning"[Title/Abstract] OR "Deep Learning"[Title/Abstract]) AND ("Uterine Cervical Neoplasms"[Mesh] OR “cervical”[Title/Abstract] OR “cervix”[Title/Abstract]) AND ("Lymphatic Metastasis"[Mesh] OR "Lymphatic Vessels"[Mesh] OR "lymphatic invasion"[Title/Abstract] OR "lymphovascular invasion"[Title/Abstract] OR "lymphatic vessel invasion"[Title/Abstract] OR "lymphatic permeation"[Title/Abstract] OR "lymphatic metastasis"[Title/Abstract] OR "lymphovascular permeation"[Title/Abstract] OR "LVI"[Title/Abstract] OR "lymphatic spread"[Title/Abstract] OR “LVSI”[Title/Abstract]) |
| Embase | ('artificial intelligence'/exp OR 'machine learning'/exp OR 'deep learning'/exp OR ‘Artificial Intelligence’:ab,ti OR ‘AI’:ab,ti OR ‘Machine Learning’:ab,ti OR ‘Deep Learning’:ab,ti) AND ('uterine cervix tumor'/exp OR ‘cervical’:ab,ti OR ‘cervix’:ab,ti) AND ('lymph node metastasis'/exp OR 'lymph vessel metastasis'/exp OR 'lymphatic invasion':ti,ab OR 'lymphovascular invasion':ti,ab OR 'lymphatic vessel invasion':ti,ab OR 'lymphatic permeation':ti,ab OR 'lymphatic metastasis':ti,ab OR 'lymphovascular permeation':ti,ab OR 'LVI':ti,ab OR 'lymphatic spread':ti,ab OR ‘LVSI’:ab,ti) |
| Web of Science | ((TS=("Uterine Cervical Neoplasms" OR “cervical” OR “cervix”)) AND TS=("Artificial Intelligence" OR "Machine Learning" OR "Deep Learning" OR "AI" OR "Machine Learning" OR "Deep Learning")) AND TS=(“lymph vessel metastasis” OR "Lymphatic Vessels" OR "lymphatic invasion" OR "lymphovascular invasion" OR "lymphatic vessel invasion" OR "lymphatic permeation" OR "lymphatic metastasis" OR "lymphovascular permeation" OR "LVI" OR "lymphatic spread" OR “LVSI”) |
| Cochrane Library | (Orthognathic Surgery OR Orthognathic Surgical Procedures OR Jaw Surgery OR Jaw Surgeries OR Maxillo-Mandibular Surgery OR Maxillofacial Orthognathic Surgery OR Bimaxillary OR two-jaw OR double-jaw OR surgical orthodontic):ti,ab,kw AND (sequencing OR sequence OR maxilla-first OR mandible-first):ti,ab,kw |
